# Logistic function explains the growth dynamics of different types of shoots of four olive cultivars grown in a super high-density orchard

**DOI:** 10.3389/fpls.2025.1542816

**Published:** 2025-06-19

**Authors:** Leonardo Costanza, Diego Maria Pinto, Luigi Pinto, Gaetano Alessandro Vivaldi, Salvatore Camposeo

**Affiliations:** ^1^ Department of Soil, Plant and Food Science, University of Bari Aldo Moro, Bari, Italy; ^2^ Istituto di Analisi dei Sistemi ed Informatica “Antonio Ruberti”, Consiglio Nazionale delle Ricerche, Rome, Italy; ^3^ BF Research, Life Science and Technology Department, Jolanda di Savoia, Italy

**Keywords:** vigor, self-rooted, proleptic, sylleptic, adventitious, crop load

## Abstract

**Introduction:**

The olive tree (*Olea europaea* L.) has cultural, economic, and environmental importance in the Mediterranean region. In the last two decades, olive cultivation has shifted from low-density to super high-density (SHD) planting systems. These systems are characterized by narrow hedgerows of low-vigor, early-bearing cultivars, allowing full mechanization. However, limited information are available on shoot growth dynamics of the olive tree under the SHD system. This study aimed to investigate the shoot growth dynamics of four olive cultivars (‘Arbequina’, ‘Coratina’, ‘Frantoio’, and ‘Urano’) by modeling the elongation of different shoot types (apical proleptic, lateral proleptic, sylleptic) under SHD conditions.

**Methods:**

A four-year field study was conducted on four olive cultivars (‘Arbequina’, ‘Coratina’, ‘Frantoio’, and ‘Urano’) grown in an SHD orchard under Mediterranean climate. Apical proleptic, lateral proleptic, sylleptic, and adventitious shoots were monitored. Logistic regression was applied to model shoot elongation, and statistical analyses were conducted to assess the influence of cultivar, shoot type, and year. Moreover, the effect of crop load and temperature on shoot growth was also evaluated.

**Results and Discussion:**

No significant difference was shown between the type of shoot and cultivar. Results indicate that single-phase logistic growth was the most common pattern, except for the lateral proleptic shoots of ‘Coratina’ and adventitious of ‘Urano’, where a second vegetative flush occurred. No correlation of Growing Degree Days with the shoot growth was observed. As confirmed in previous studies, crop load showed a negative influence on shoot elongation. Particularly for Arbequina’s adventitious shoots, Coratina’s and Frantio’s sylleptic shoots and Urano’s lateral proleptic, this trend was observed. This evidence showed the potential competition between the reproductive and vegetative cycle for assimilates. To our knowledge, this is the first report addressing the vegetative growth dynamics of four different shoot types of four distinct olive cultivars with different vigor in an SHD system. These findings are essential for optimizing cultivar-specific agricultural strategies (e.g. canopy management and irrigation) to achieve an optimal yield and sustainable cultivation. Future research will explore the vegetative growth dynamics, including other factors such as trunk diameter, Leaf Area Index, and water stress indices.

## 1 Introduction

In the Mediterranean area, the olive tree (*Olea europaea* L., 1753) is the most cultivated fruit tree species, integrated with the culture and economy (Garofalo et al., 2024a; Costanza et al., 2024). During the past 20 years, olive growing has been shifting from traditional low-density to super high-density (SHD) orchards, representing an innovative planting system (Guerrero-Casado et al., 2021). It is characterized by a continuous narrow hedgerow of trees of low-vigor and early bearing cultivars, planted at a density between 1,500 and 2,000 trees ha^-1^ (Rallo et al, 2013; Connor et al., 2014; Tous et al., 1999; Rallo et al., 2007; Rosati et al., 2018a). Compared to the traditional system, SHD enables fully mechanized harvesting and pruning techniques to reach a high crop level, preserving environmental sustainability (Russo et al., 2015; Pellegrini et al., 2016; Taguas et al., 2021; Camposeo et al., 2022). Furthermore, to obtain an optimal long-term performance, the cultivar plays a key-role (Díez et al., 2016; Rosati et al., 2024); some olive cultivars are suitable for this training system, due to their limited tree vigor. The main cultivar used for SHD is ‘Arbequina’ and ‘Arbosana’ (Tous et al., 2014); recently other cultivar have been introduced like ‘Oliana’ and ‘Lecciana’ (Camposeo et al., 2021). Moreover, the cultivar ‘Urano’ (patented as ‘Tosca’) showed interesting features fitting for SHD oliveculture, such as low vigor and early bearing (Camposeo et al., 2008; Vivaldi et al., 2015). Traditional cultivars as ‘Coratina’ and ‘Frantoio’ could be “*escape*” from the concept of a small canopy-low vigor due to their genetic (Rosati et al., 2024; Paoletti et al., 2021). To know if a cultivar is suitable for the SHD planting system, characterizing the architectural traits is essential (Rosati et al., 2013; Bufacchi et al., 2024). Analyzing plant architecture is essential for understanding growth patterns, branching structures, and potential yield, as well as the improvement of crop models and canopy/root management strategies (Maldera et al., 2024a; Paoletti et al., 2023), including specific parameters like growth, branching, morphological differentiation of axes, and the apical vs. lateral position of reproductive structures (Rosati et al., 2013; Carella et al., 2022). According to the time of woody bud breaking on shoot, two types can be distinguished: proleptic, if they come from resting buds; sylleptic, if they derive from steady buds. Conversely, the adventitious shoots derive from latent buds imbedded in the wood stimulated by different factors as: injury, hormonal status, and accumulation of carbohydrates in the parenchyma cells of sapwood (Lanner, 2002; Del Tredici, 2001; Climent et al., 2004). For adult olive trees in Mediterranean climates, shoot elongation occurs mostly from March to July, but it is largely affected by environmental conditions, tree age, phenological stage, and crop load (Rallo, 1998; Bandino and Dettori, 2001). On the other hand, young olive trees are characterized by rapid growth rates, reaching full size at ten years of age (Bongi and Paliotti, 1994). Studies on vegetation development show that olive tree growth is mainly controlled by temperature, water availability, and competition for assimilates (Benlloch-González et al., 2024; Cinosi et al., 2024; Iglesias et al., 2024; Benlloch-González et al., 2018; Miserere et al., 2018; Palese et al., 2010; Portarena et al., 2024; Rallo et al., 2016). Rallo et al., 1994 reported temperature as a main factor controlling bud development and shoot elongation, which may accelerate or decelerate growth rates at any stage of growth. The logistic equation is the best-known used to describe biological growth processes (Verhulst, 1838; Yin et al., 2003). Several models of shoot growth have been proposed for deciduous fruit tree crops such as peach (Kervella et al., 1995), apple (Rejeb, 1997), and almond (Esparza et al., 1998).

This study aims to evaluate the growth dynamic of four types of shoots in four olive cultivars with different vigor, under a SHD system, implementing a logistic model; in addition, the effect of type of shoot, cultivar, year, exposure, growing degree days (GDD), and crop load on growth dynamics was considered. While considering several classes of function for shoots elongation modeling, human interpretable model explainability is one of the main metrics of model class choice; according to this approach, significantly easier to-explain and interpret functions are preferred to high computational complexity and fitting accuracy of black-box modeling (Garofalo et al., 2024b). Models related to the machine learning framework are indeed not considered for the autoregressive target model of this work. Nevertheless, research effort is dedicated to model selection to minimize regression error while guaranteeing generalization properties. Since the time-varying environmental conditions are known to influence shoots growth, and nature related growth phenomena may be well described by the class of logistic functions, these are considered to address the research objective and described in Material and Methods section. The same section includes the description of the dataset used to test such a class of functions as a proper modeling approach for shoot elongation, along with relevant statistical analysis.

## 2 Materials and methods

### 2.1 Olive orchard characteristics

**Figure 1:**
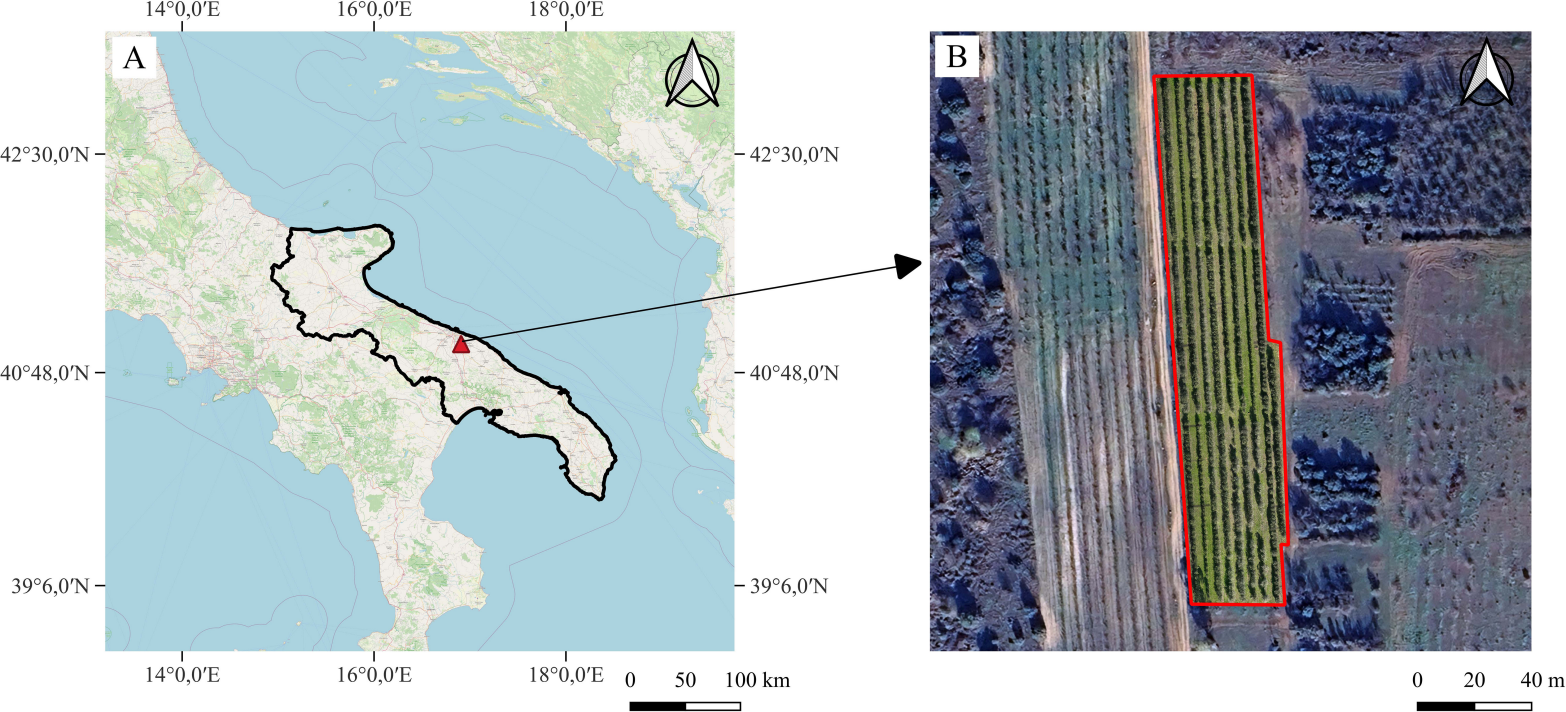
Geolocation **(A)** and orthophoto **(B)** of the experimental SHD olive orchard located at the University of Bari experimental farm in Valenzano (Openstreetmap.org, 2024; Map data ^©^2015 Google).

The study was carried out in an olive grove located at the experimental farm of the Department of Agricultural and Environmental Sciences at Valenzano (**Figure 1A**) (Bari, Southern Italy – 41°01’N; 16°45’E; 110 m a.s.l.), with a sandy clay soil (sand, 630 g·kg^−1^; silt, 160 g·kg^−1^; clay, 210 g·kg^−1^) classified as a Typic Haploxeralf (USDA) or Chromi-Cutanic Luvisol (FAO). The site was characterized by a typical Mediterranean climate, with an average annual rainfall of 603 mm, two-thirds concentrated from autumn to winter, and a long-term average annual temperature of 16.4°C (regional agro-meteorological service - ARIF). The olive grove was planted in spring 2006 with self-rooted plants; the trees were trained according to the central leader system and spaced 4.0 m x 1.5 m (1,667 trees ha^-1^) with North-South orientation according to the SHD planting system (**Figure 1B**). Drip irrigation was managed by restoring the 100% of Etc. Nutrition, weed management, and disease control were implemented in the same manner for all cultivars (Camposeo and Vivaldi, 2011; Camposeo et al., 2013). The first significant yield was recorded in autumn 2008. Canopy management was started in winter 2009–2010 with annual manual thinning. Mechanical pruning started in 2012 with topping (240 cm height), hedging (50 cm from the central stem), and trimming (60 cm from the ground) using tractor-mounted machines. Manual thinning removed branches over 3–4 cm in diameter (Vivaldi et al., 2015). In all cases, the cultivars were subjected to the same pruning treatment each year.

### 2.2 Experimental design

The study was conducted on four self-rooted cultivars with different vigor: ‘Urano’, ‘Arbequina’, ‘Coratina’ and ‘Frantoio’ (ascending order). The measurements were taken in four years (2009–2012) respectively at the 4^th^, 5^th^, 6^th^, 7^th^ year after planting (YAP), starting from May to November. A randomized block experimental design was adopted; for each cultivar, the measurements have been replicated on three blocks of three trees and the observations were carried out on all trees of each block. Before woody bud breaking, two healthy well light-exposed one-year shoots per tree (East-West side of the row) were labelled (18 one-year shoots per cultivar) in the middle part of the crown. Each one-year shoots had an average length of 25–30 cm with 15–20 nodes as well. After woody buds breaking, the length of the proleptic (both apical and lateral), sylleptic and adventitious shoots developed on each one-year shoots was measured with a fortnightly frequency. Per each type of shoots, year and cultivar, the elongation of three shoots were measured.

### 2.3 Field data

Per each of field measurements, temperature and rainfall data (with hourly frequency) were retrieved by the regional Agro-meteorological service database (ARIF). Temperature (°C) data were useful to calculate Growing Degree Days index (GDD). GDD is a bioclimatic index (Badr et al., 2018; Honorio et al., 2018), based on the concept that plants grow if the air temperature exceeds a specific base temperature value for a certain period (Łysiak and Szot, 2023). To compute GDD, we used the *chillR* package of R programming language (Luedeling and Fernandez, 2022) with a base temperature threshold (T_base_) equal to 7°C as suggested by Charalampopoulos et al., 2021. **Table 1** reports the olive yield (kg · tree⁻¹) for each cultivar per year as the average productivity of the olive trees within a single row. Additionally, crop load status (“on” or “off” year) was estimated based on year-to-year variations in olive yield. **Table 2** showed the GDD values during the measurements period across different years.

**Table 1:** Olive yield (kg tree^-1^) of each cultivar from 4^th^ (IV) to 7^th^ (VII) year after planting (YAP).

	Olive yield (kg tree^-1^)			
Cultivar	2009 IV	2010 V	2011 VI	2012 VII
Arbequina	5.6	5.4	4.0	5.0
Coratina	5.1	4.5	0.6	0.5
Frantoio	1.4	3.1	0.5	1.5
Urano	2.2	2.0	2.5	2.8

**Table 2:** Growing degree days (GDD) accumulated during the shoot measurement across the study period.

Year	Doy	GDD
2009	127	597.77
	154	963.14
	183	1391.17
	216	1988.98
	252	2614.38
	281	2971.22
	313	3193.03
2010	126	618.77
	158	951.00
	181	1302.40
	214	1879.32
	246	2125.77
	278	2828.08
	308	3044.07
2011	126	521.34
	157	890.08
	187	1372.65
	214	1856.17
	250	2526.06
	277	2938.24
	307	3201.24
2012	125	639.52
	157	1001.20
	187	1550.05
	215	2090.15
	249	2718.37
	279	3170.84
	310	3504.01

### 2.4 Modeling approach

Since time-varying environmental conditions are known to influence shoot growth, it is important to account for their effects in the modeling process. Given that growth-related natural phenomena are often well described by logistic functions, we adopted this class of functions to analyze the dataset and address our research objective. According to the logistic function, the initial stage of growth is approximately exponential, then as saturation begins the growth slows to linear, and at maturity growth stops (Vandermeer, 2010). Equation 1 presents a general formula for this class of function.


(1) $$f(t)=\frac{L}{1+e^{-g(t-m)}}$$

where the growth rate (g), the upper asymptote of elongation (L) and the curve inflection point (m) that separates the early stages of growth from the asymptotic phase.

Because a relevant number of samples proved that such a function could not guarantee generalization properties, also a double logistic function is used to model such samples growth behavior. Such function is presented in Equation 2 and is able to model such two distinct phases of growth.


(2) $$f(t)=\frac{L_{1}}{1+e^{-g_{1}(t-m_{1})}}+\frac{L_{2}}{1+e^{-g_{2}(t-m_{2})}}$$

Equations 1 and 2 are used as classes of function to such regression tasks, and each set of possible parameters g, L, and m identify a specific function, thus a specific sample behavior. The estimation of such parameters, for each sample, is performed by selecting the best combination of estimators that minimize a given regression error. We considered the mean squared error (MSE), described in Equation 3 as a proper regression error.


(3) $$MSE=\frac{1}{n}+\sum_{i=1}^{n}{\left(Y_{i}+{\hat{Y}}_{i}\right)}^{2}$$

Then for each shoot sample (i.e. apical and lateral proleptic, sylleptic, adventitious), its set of parameters (g, L, m) are identified employing a proper optimization routine aimed at finding the set of parameters that minimizes (3). These parameters are saved and attached to the original dataset of shoot samples, so that each of these is also associated with its describing parameters, either with single (1) or double (2) logistic functions. A fine-tuning phase is then devoted to distinguishing if each shoot sample is better described by a single or a double logistic function. This cannot be performed considering MSE, as (2) always produce a lower regression error by empowering twice the number of modeling parameters. Therefore, we implemented a threshold-based approach presented in the following: Given the set parameters of L_1, m_1, L_2, m_2 of (2), two thresholds, m_threshold ∈ R+ and L_threshold ∈ [0,1], are used to perform this task of assigning each sample to either the class of single or double logistic functions. This phase is performed by computing the following:

- Evaluate if m_2 - m_1 > m_threshold
  ○if True:
    ▪compute L_tot = L_1 + L_2 (i.e., total elongation of the two growth phases)
    ▪evaluate if L_2 - L_1 > L_threshold * L_tot (i.e., a percentage of L_tot)

- if True:
  ○Shoot sample belong to the double logistic class

- if False:
  ○Shoot sample belong to the single logistic class

○if False:
  ▪Shoot sample belong to the single logistic class

This routine is then applied to the shoots samples’ dataset (augmented with regression parameters estimators) leveraging m_threshold = 1 and L_threshold = 0.1.

### 2.5 Statistical analysis

To analyze the growth dynamics of olive shoots, logistic curves were fitted to the dataset by estimating the parameters L (asymptotic maximum length), m (growth midpoint), and g (growth rate) for each shoot through the minimization of an error function. The goodness of fit of the logistic models to the experimental data was assessed using MSE and the coefficient of determination (R²). The results indicate that the logistic function provides a highly accurate representation of shoot growth, with MSE values approaching zero in most cases and R² values consistently close to 1. The goodness of such regression fits can be appreciated through the analysis of residuals, which is presented in the **Supplementary Figures S1**, **S2**. Specifically, **Supplementary Figures S1** provides box plots of the residuals for each of the seven time points, corresponding to the seven measurements of shoot length. The distribution of residuals across different time points highlights the consistency of the model’s performance across all stages of shoot growth. Additionally, **Supplementary Figures S2** showed a Q-Q plot of the residuals, which further supports the assumption of normality and indicates an overall well-behaved residual distribution. The alignment of residuals with the theoretical quantiles suggests that the logistic model adequately captures the growth dynamics of the olive shoots. Furthermore, **Supplementary Figures S3** displays the logistic fit of the model along with the actual observed data points for seven individual shoots. This visualization allows for a direct assessment of how the fitted curves represent the real data, reinforcing the appropriateness of the logistic model for describing shoot growth dynamics.

Once the logistic parameters were estimated for each sample, a Welch-Test was conducted to detect any significant differences in these parameters across groups. Since in some cases, Kolmogorov-Smirnov and Hartley tests indicated that the dataset violated both the normality and the heteroscedasticity, a non-parametric alternative to ANOVA was applied. The Kruskal-Wallis test was chosen as the appropriate method for comparing the differences between the median values of the parameters L, m, and g across groups.

## 3 Results and discussion

### 3.1 Vegetative growth dynamics

Despite no statistical differences was observed between cultivar and type of shoot (p > 0.05), the tendency of shoot growth dynamic, in irrigated conditions, was expressed by a single logistic function as shown in **Table 3**. This is because a second rhythmic growth pattern is more frequent in rainfed conditions (Mezghani et al., 2008). This pattern was particularly pronounced in the apical proleptic, lateral proleptic, and sylleptic shoots following the **Equation 1**. ‘Frantoio’ showed a 100% of single logistic model in both apical proleptic and sylleptic shoots. On the contrary, ‘Urano’ showed a slight tendency for double logistic growth, in the lateral proleptic (20%) and adventitious (21%) shoots. ‘Arbequina’ showed a high percentage of single logistic growth between all studied shoot, with the adventitious shoots that had the highest percentage. ‘Coratina’ showed a distinct pattern, with both single and double logistic growth model; adventitious shoots represented the shoot type with the highest incidence of double logistic growth (21%), like ‘Urano’. In the northern hemisphere, olive trees typically experience two vegetative growth flushes: the main one occurs between March and mid-July, while the second flush happens from September to mid-October unless water availability is not a limiting factor (Benlloch-González et al., 2019).

**Table 3:** Percentage of single (on the left) and double (on the right) shoots growth dynamic, obtained by logistic regression, for each cultivar and type of shoot.

Cultivar	Type of shoot			
	Apical Proleptic	Lateral Proleptic	Sylleptic	Adventitious
Arbequina	74–26 n.s.	96–4 n.s.	91–9 n.s.	100–0 n.s.
Coratina	87–13 n.s.	94–6 n.s.	87–13 n.s.	79–21 n.s.
Frantoio	100–0 n.s.	94–6 n.s.	100–0 n.s.	93–7 n.s.
Urano	86–14 n.s.	80–20 n.s.	89–11 n.s.	79–21 n.s.

**Figure 2:**
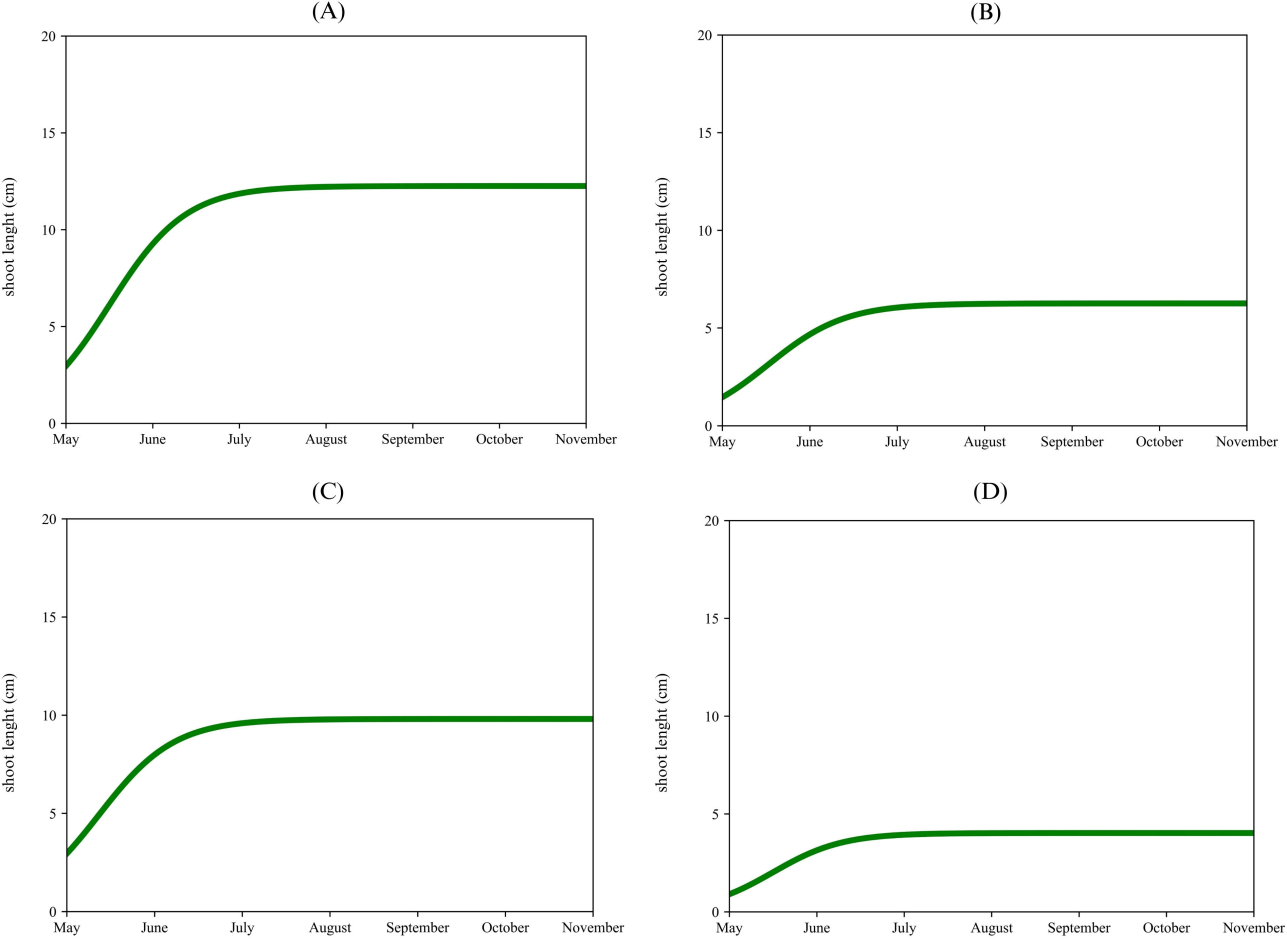
Single logistic growth curve of ‘Arbequina’ per type of shoot: apical proleptic **(A)**, lateral proleptic **(B)**, sylleptic **(C)**, adventitious **(D)**.

**Figure 3:**
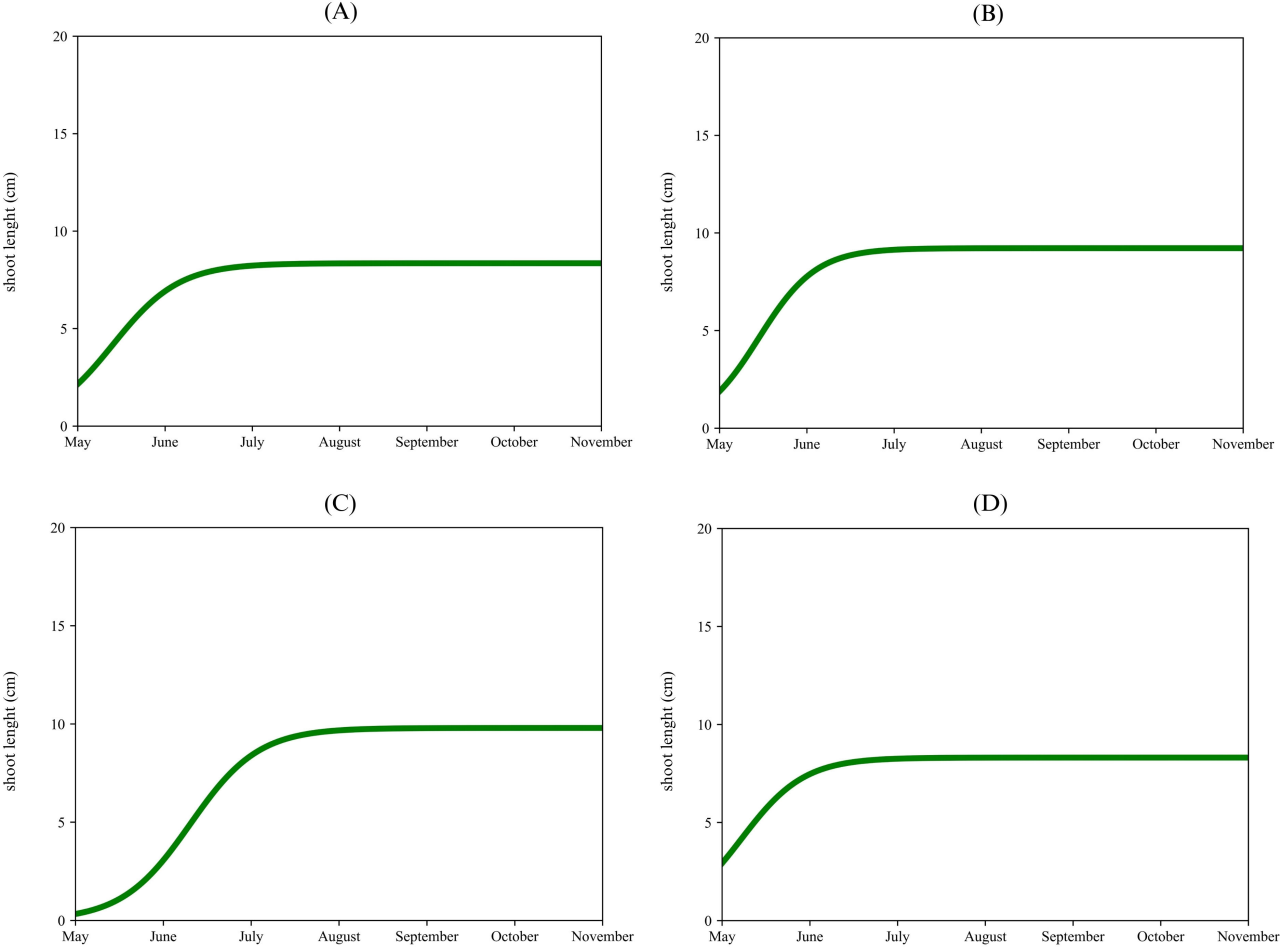
Single logistic growth curve of ‘Coratina’ per type of shoot: apical proleptic **(A)**, lateral proleptic **(B)**, sylleptic **(C)**, adventitious **(D)**. n.s., not significant.

**Figure 4:**
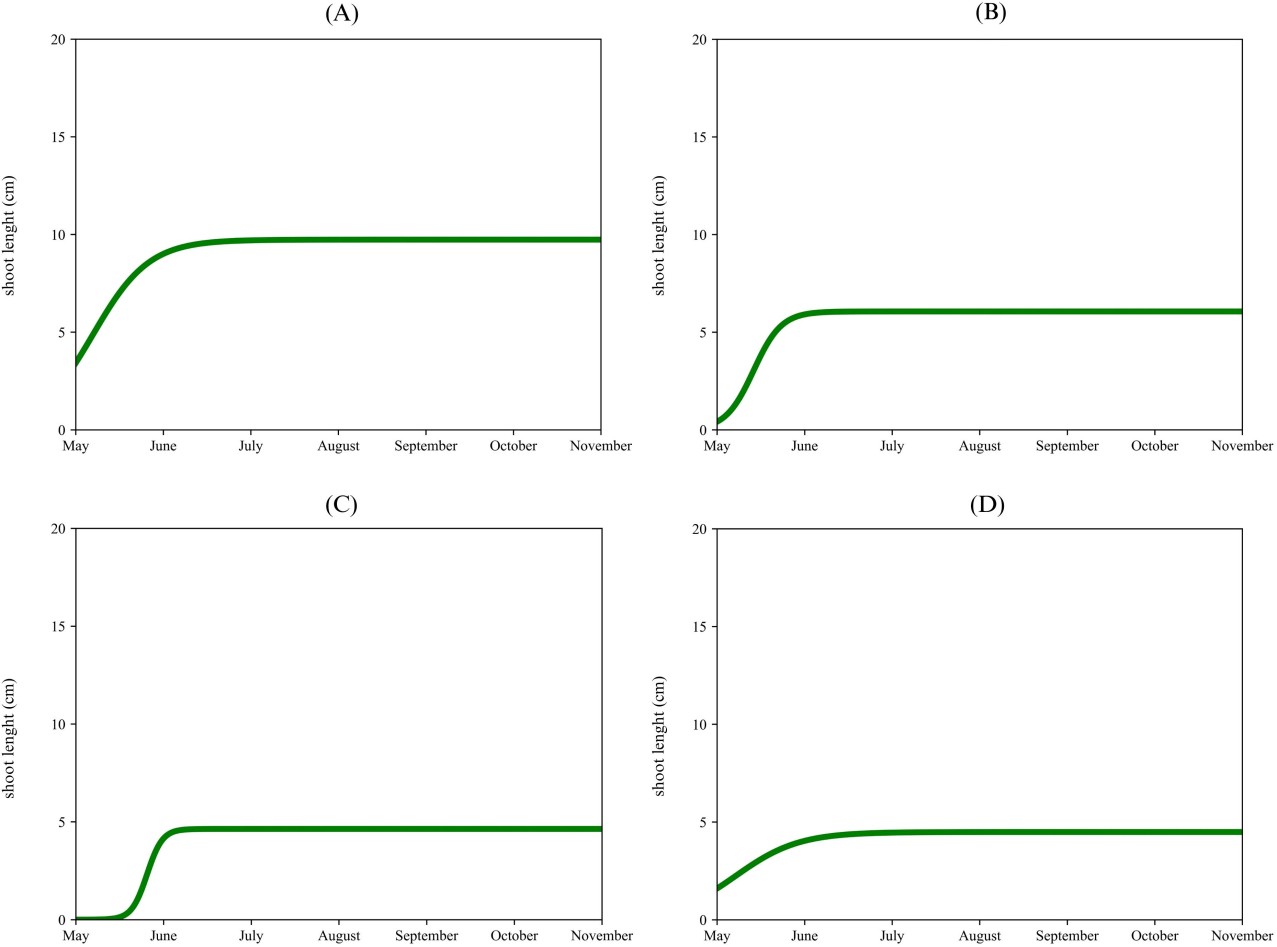
Single logistic growth curve of ‘Frantoio’ per type of shoot: apical proleptic **(A)**, lateral proleptic **(B)**, sylleptic **(C)**, adventitious **(D)**.

The apical proleptic shoots (**Figure 2A**) of ‘Arbequina’ showed a rapid growth phase from May to the middle of June, reaching a plateau of 12 cm during early July. The lateral proleptic shoots (**Figure 2B**) showed a similar growth pattern but with a slightly shorter final length, reaching a plateau of 6 cm. The sylleptic shoots (**Figure 2C**) also show rapid growth from May to June, stopping at 9 cm. Lastly, the adventitious shoots (**Figure 2D**) exhibited the slowest and shortest growth (4 cm) respect to the previous shoots. For ‘Coratina’, the apical proleptic shoots (**Figure 3A**) demonstrated a rapid growth phase from May to June, reaching a plateau of 8 cm in July. The lateral proleptic shoots (**Figure 3B**) showed a similar trend with a plateau of 8 cm. For the sylleptic shoots (**Figure 3C**) was observed a rapid growth phase, reaching a plateau at 9 cm in July. The adventitious shoots (**Figure 3D**) showed the slowest growth, stopping at 7 cm. The growth curve for the apical proleptic shoot of ‘Frantoio’ (**Figure 4A**) started with a rapid growth in May, reaching a final length of 9 cm in July. The lateral proleptic (**Figure 4B**) shoots showed slight delay in its starting growth, achieving the plateau phase in July with a length of 6 cm. The sylleptic shoots (**Figure 4C**) demonstrated a more restrained growth pattern, reaching a final length of 5 cm in June. The adventitious shoots (**Figure 4D**) followed a similar pattern of the sylleptic ones, stabilizing around 5 cm in June. These growth patterns indicated a rapid starting vegetative growth phase followed by a plateau for all shoots, where the apical and lateral proleptic showed the major length compared to sylleptic and adventitious ones. This suggests that apical and lateral proleptic shoots might play an essential role in the vegetative cycle of ‘Frantoio’, influencing its architectural and reproductive characteristics (Paoletti et al., 2021). The equal stabilization of vegetative growth cycle between all shoot types in the middle of the year, highlights the influence of genetic factors in this cultivar (Benlloch-González et al., 2019).

**Figure 5:**
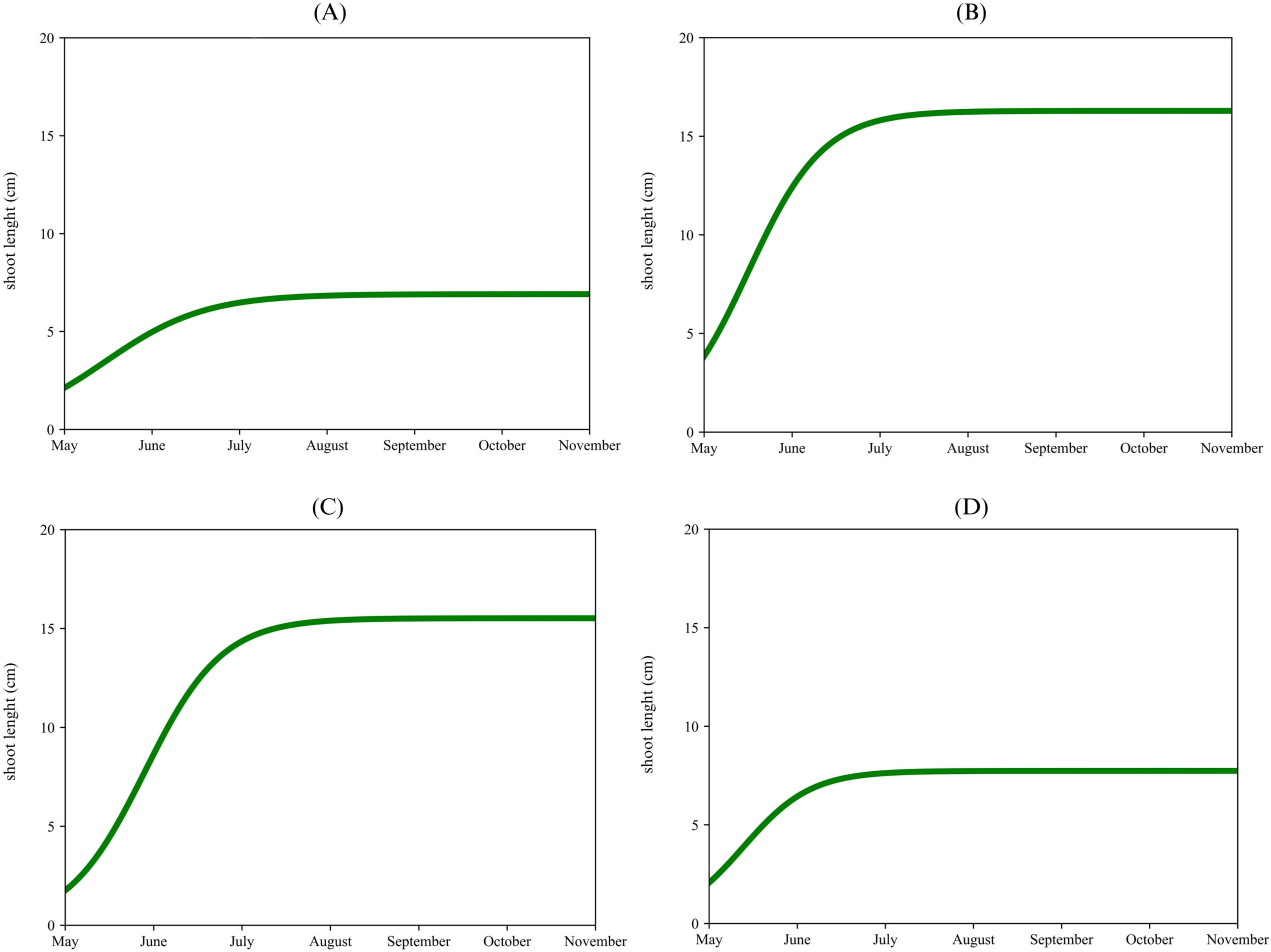
Single logistic growth curve of ‘Urano’ per type of shoot: apical proleptic **(A)**, lateral proleptic **(B)**, sylleptic **(C)**, adventitious **(D)**.

In the apical proleptic shoots of ‘Urano’ (**Figure 5A**), was observed an initial gradual increase in May, reaching a plateau of 7 cm in July keeping this length until November. The lateral proleptic shoots (**Figure 5B**) showed a quick growth phase from May to June, reaching a final length of 16 cm in the middle of June until the end of the observed period. Sylleptic shoots (**Figure 5C**) showed a similar growing phase respect to the previous one, achieving a final length of 15 cm in July. Adventitious shoots (**Figure 5D**), started with a gradual growth phase, reaching a plateau of 7 cm in June. Different vegetative growth dynamics per shoot type and cultivar, suggested a different response to the hormonal stimuli (Proietti and Tombesi, 1996; Dag et al., 2010; Fernández et al., 2015). Examining the chronology of the events between the first and second phase of shoots growth provides suggestions about the competition between these two processes, which may involve in a shifting of this growth flushes. Mezghani et al., 2008 observed the influence of several factors between the starting and interruption of these flushes. Our results support the hypothesis of a causal link between the onset of primary growth and the end of secondary growth, which can be attributed either to changes in auxin ratios (Digby and Wareing, 1966a, b) or other source-sink dynamics (Castrodiez et al., 1998; Costes et al., 2000). Castillo-Llanque and Rapoport (2011) found that trees with greater nutrient availability (non-bearing trees in their study) produced more sylleptic shoots, promoting node formation in the vegetative sections of 1-year-old shoots and sylleptic shoots. Additionally, the growth rate of the parent shoot can influence whether axillary buds develop into sylleptic shoots (Costes et al., 2006), while the presence of fruit can impact the number of sylleptic shoots produced during the growth of 1-year-old shoots (Castillo-Llanque and Rapoport, 2011).

**Figure 6:**
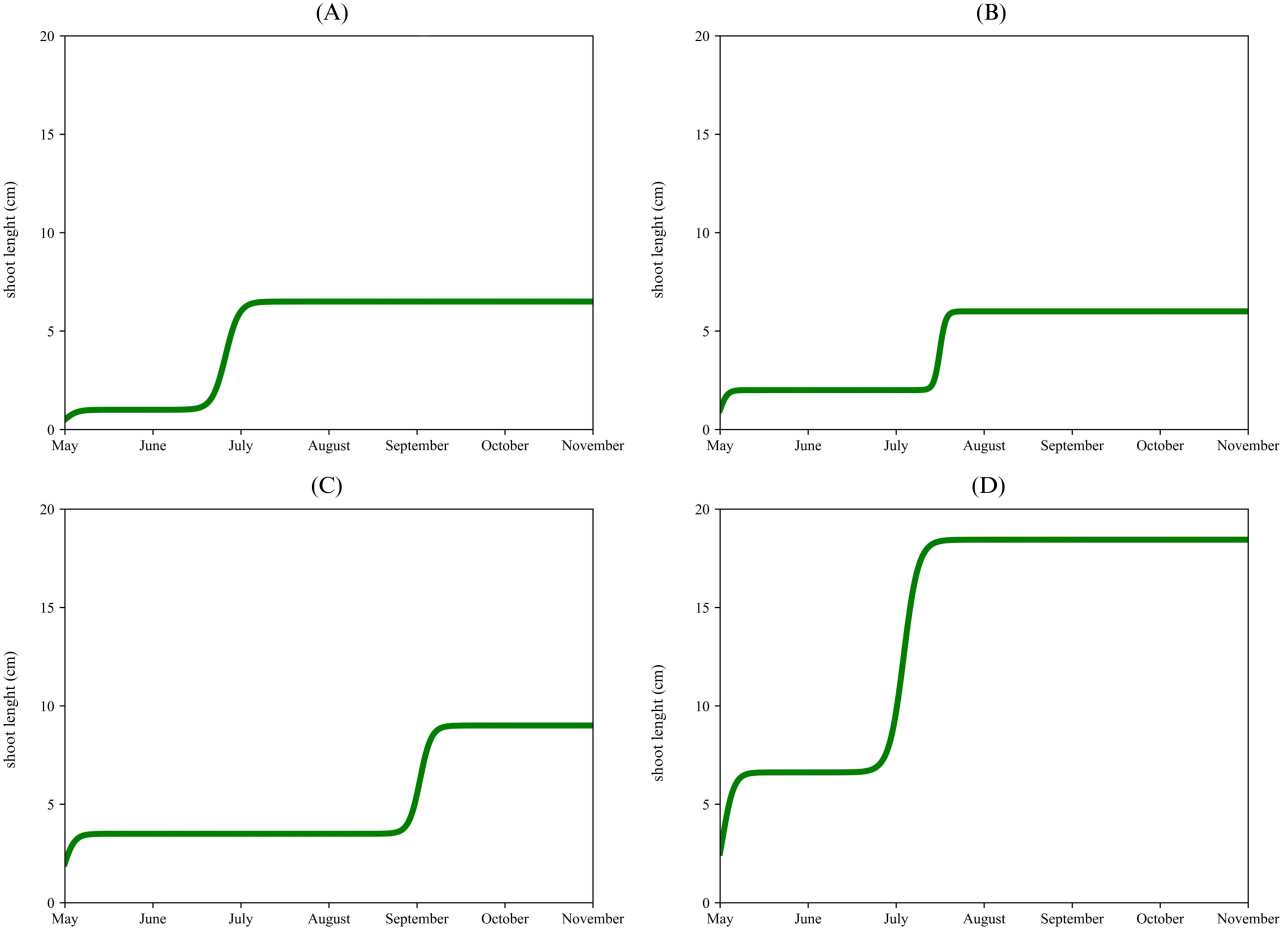
Double logistic growth curve of Arbequina’s apical proleptic shoot **(A)**, Coratina’s lateral proleptic **(B)**, Urano’s lateral proleptic **(C)**, and Urano’s adventitious **(D)**.

**Figure 6** illustrated the cultivar and shoots where a second flush growth was also observed. In the apical proleptic shoots of ‘Arbequina’ (**Figure 6A**), a second vegetative growth flush (**Equation 2**) was observed in the middle of June, reaching a final length of 7 cm, until the end of the observed period. Similarly, the lateral proleptic shoots of ‘Coratina’ (**Figure 6B**) showed a second growth stage at the beginning of July, with a plateau of 6 cm. For the Urano’s lateral proleptic shoots (**Figure 6C**) was noticed a second vegetative flush between the end of August and the beginning of September e, reaching a final length of 9 cm. The adventitious shoot of ‘Urano’ (**Figure 6D**) showed a different pattern respect to the previous ones. After an initial vegetative growth phase that stopped at the end of June (plateau of 6 cm), a fast second vegetative flush was observed at the beginning of July, reaching a final growth of 18 cm in the middle of July. A common model of olive tree shoot elongation describes two active growth phases: the main one in spring, before blooming, and a secondary, less significant phase in early autumn, with a summer dormancy in between (Lavee, 2007). Our data confirmed an opposite tendency with a secondary vegetative flush during the summer season, except to the lateral proleptic shoots of ‘Urano’ cultivar. These second vegetative growth flushes underscored the importance cultivar choice in higher planting densities (Strippoli et al., 2013). from an architectural point of view. No influence of exposure on each type of sprouts among the four cultivars, was observed.

### 3.2 Cultivar and shoot influence

**Figure 7:**
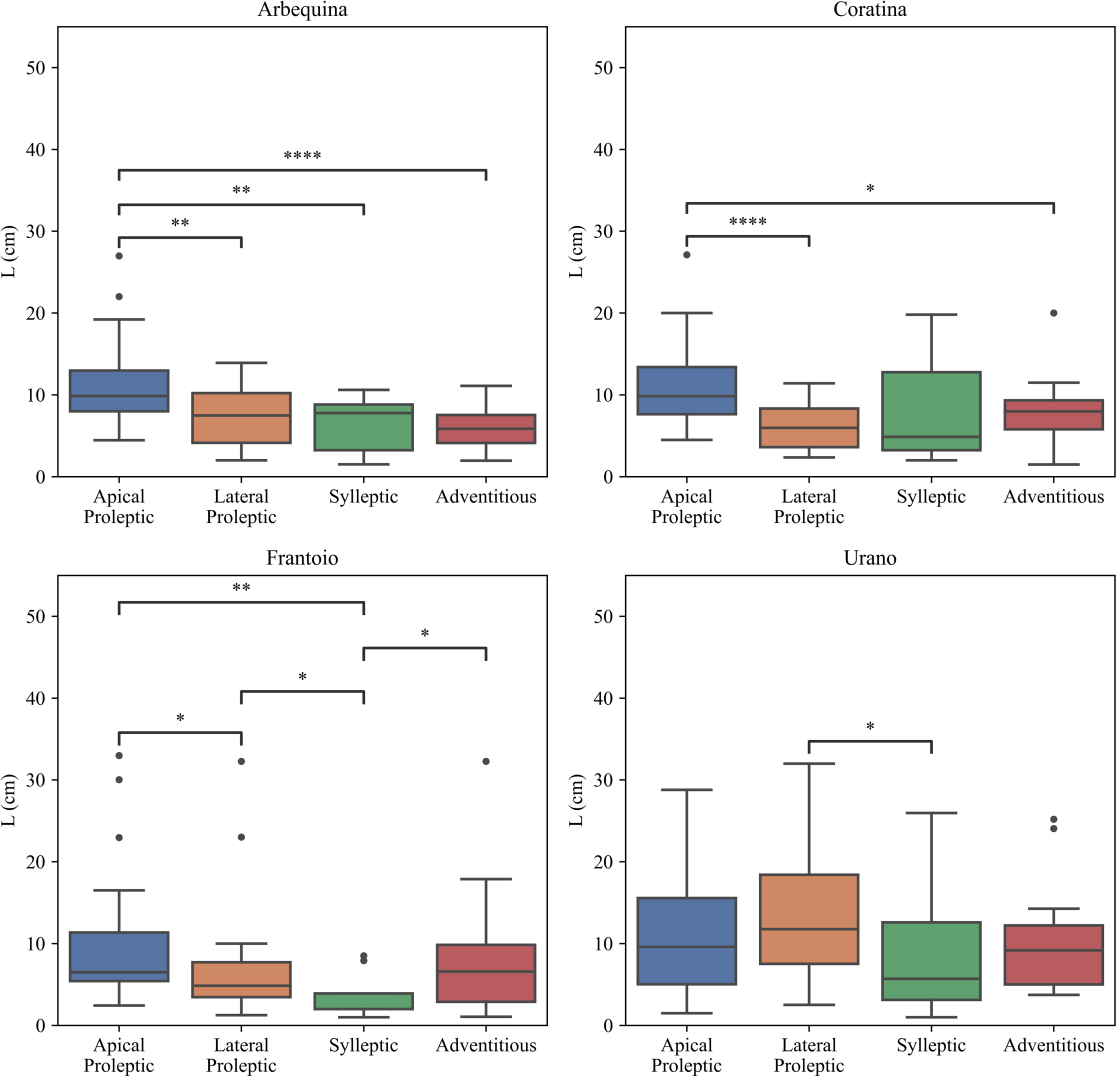
Effect of cultivar and type of shoots on shoot growth (L). The statistical analysis was conducted using the Kruskal-Wallis test. Significant differences between groups are indicated by asterisks (*p < 0.05, **p < 0.01, ***p < 0.001, ****p < 0.0001).

The apical proleptic shoots of ‘Arbequina’ showed the major final growth length followed by lateral proleptic, sylleptic, and adventitious ones (**Figure 7**). Furthermore, apical proleptic shoots showed a significant differences with lateral proleptic (p < 0.01), sylleptic (p < 0.01), and adventitious (p < 0.0001). This indicates a hierarchical vegetative growth of apical proleptic shoots within the ‘Arbequina’ cultivar. For ‘Coratina’ sylleptic shoots was observed a significant difference for both lateral proleptic (p < 0.0001) and adventitious shoots (p < 0.05). However, no significant differences were observed between apical proleptic and the others shoots. In the ‘Frantoio’ cultivar apical proleptic shoots had the greater length, significantly respect to the lateral proleptic (p < 0.01) sylleptic ones (p < 0.05), and adventitious (p < 0.05). This suggests that the apical proleptic and adventitious shoots of ‘Frantoio’ cultivar were favored in terms of vegetative growth, probably due to the genetic or physiological factors. Lastly, in the ‘Urano’ cultivar, statistical differences were observed between lateral proleptic and sylleptic shoot (p < 0.05). No differences were noticed among the other shoot types, indicating a uniform vegetative growth pattern.

**Figure 8:**
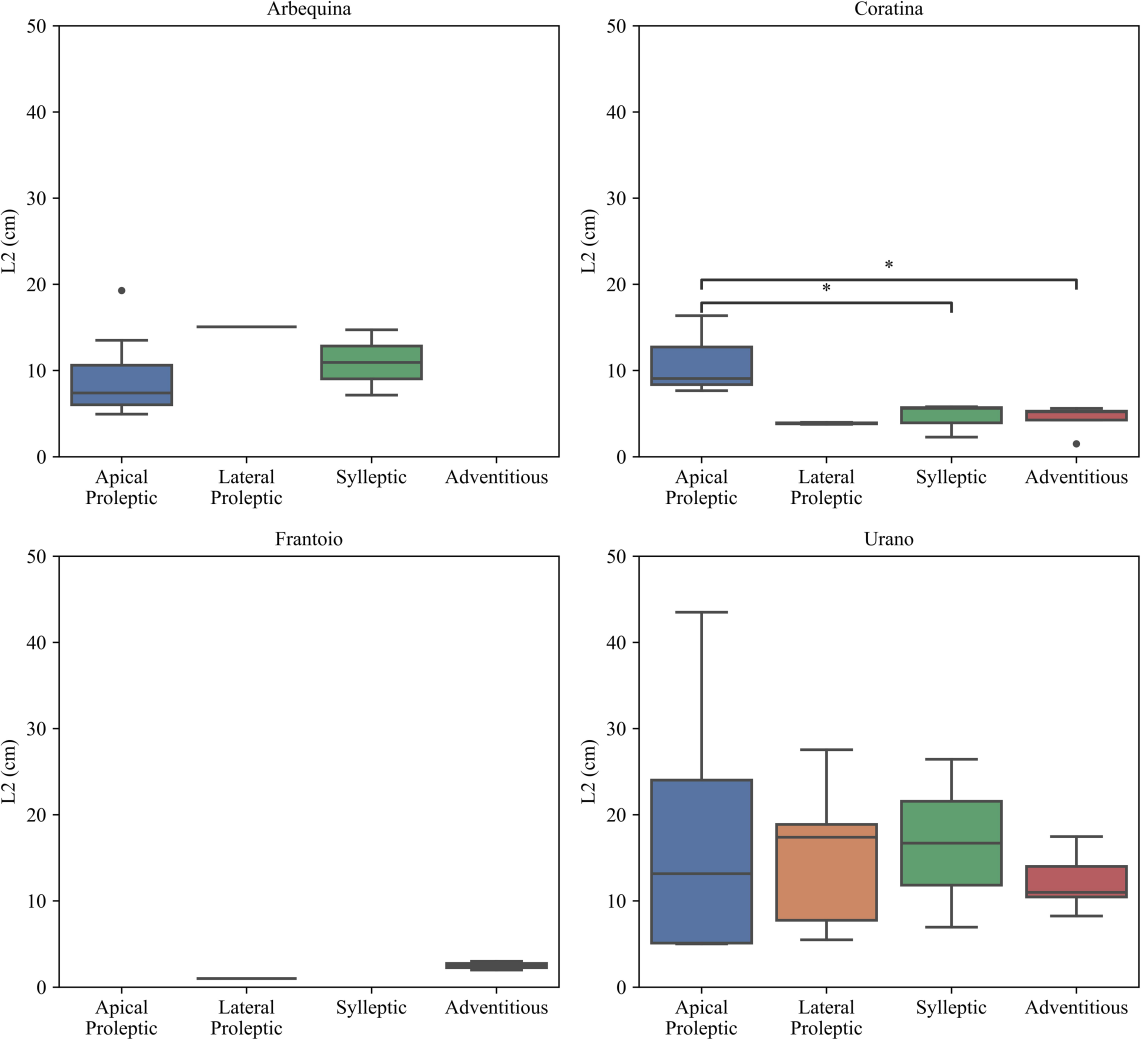
Effect of cultivar and type of shoots on second shoot growth phase (L2). The statistical analysis was conducted using the Kruskal-Wallis test. Significant differences between groups are indicated by asterisks (*p < 0.0).

The effect of cultivar and type of shoot in the second vegetative flush scenario was illustrated in **Figure 8**. For the four studied shoot types of the cultivar ‘Arbequina’, no statistical difference emerged among them. For ‘Coratina’ cultivar was observed a significant difference between apical proleptic shoots and sylleptic (p < 0.05). Final length of second flush growth of adventitious shoots were significantly different from apical proleptic ones (p < 0.05). The cultivar ‘Frantoio’ and ‘Arbequina’ shows no statistically significant difference between the observed shoots. Lastly, ‘Urano’ presented a balanced distribution of L2 values across all shoot types but with no statistical difference. Strippoli et al. (2013) report that the apical proleptic shoot elongation in ‘Arbequina’ stopped early, around the fruit set (end of May - beginning of June) phase, without a subsequent growth phase. On the contrary, ‘Coratina’ showed a second flush growth pattern, after the pit hardening (mid-July to August) phase, which aligns with the general model. However, our results were agreed with this general model for ‘Arbequina’, ‘Coratina’, and ‘Urano’ cultivar.

These results were in line with Rosati et al., 2013 that indicate how different olive cultivars have different architectural traits. To our knowledge, this is the first report about the growth dynamics of four shoot types of four different olive tree cultivar with three different levels of vigor (low, medium, and high) under a new cropping system as SHD (Rosati et al., 2024).

### 3.3 Year influence

**Figure 9:**
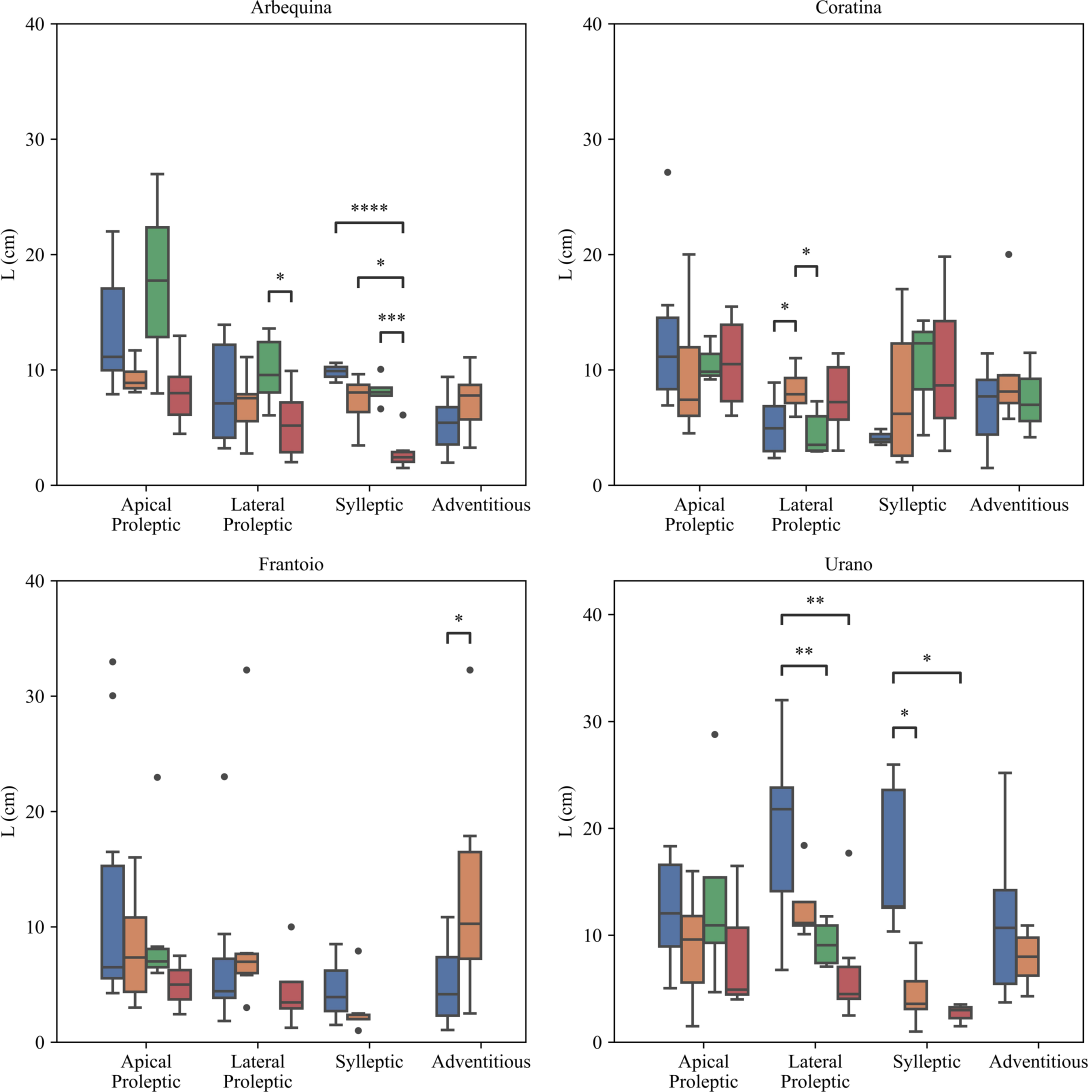
Effect of the year on shoot growth (L). Blue, orange, green, and red indicate 2009, 2010, 2011, and 2012, respectively. The statistical analysis was conducted using the Welch test. Significant differences between groups are indicated by asterisks (*p < 0.05, **p < 0.01, ***p < 0.001, ****p < 0.0001).

The year influence on shoot growth per type of shoots and cultivar was shown in **Figure 9**. ‘Arbequina’ showed a significant year-to-year variability, particularly in sylleptic shoots, between 2009 and 2012 (p < 0.0001). On the other hand, lateral proleptic shoots in 2011 had significantly greater than 2012 (p < 0.05). For ‘Coratina’, the lateral proleptic shoots showed a significant growth variability; in the 2010 these shoots showed a higher length respect to 2009 and 2010 (p < 0.05). In ‘Frantoio’ cultivar only for adventitious shoots was noticed a significant growth in 2010 respect to 2009 (p < 0.05). ‘Urano’ showed the most year-to-year vegetative growing variation: lateral proleptic shoots had a significantly greater vegetative growth in 2009 respect to 2011 and 2012 (p < 0.01). Sylleptic shoots also showed significant differences (p < 0.05), with a final length greater in 2009 compared to 2010 and 2012. The reduction in vegetative growth during fruit-bearing years is attributed to the competition for assimilates between shoots and fruits. This phenomenon was observed not only in olive trees (Connor and Fereres, 2005), but also in other fruit trees such as: apricot, avocado, peach, and pistachio (Costes et al., 2000; Salazar-García et al., 1998; Berman and DeJong, 2003; Stevenson and Shackel, 1998). An opposite trend was observed in pistachio, where Stevenson et al. (2000) reported a predominance of short shoots and a decreasing number of longer shoots in both bearing and non-bearing years.

### 3.4 Crop load and environmental influence

Crop load had a significant impact on shoot growth, reducing it (Liang et al., 2020). Olive trees exhibit a pronounced alternate bearing pattern, with alternating “on” and “off” years (Lavee, 2007). The trees regulate the source allocation, as well as the growth of the roots and canopy (Lodolini and Neri, 2012; Smith and Samach, 2013). The endogenous and exogenous factors influence on alternate bearing in olive trees can be genetic, environmental, nutritional, hormonal, or agronomic (Baktir et al., 2004; Fernández-Escobar et al., 2004; Lavee, 2007); these factors may interact either at the whole-tree level or locally at the level of individual shoots (Fichtner and Lovatt, 2018; Rosati et al., 2018; Rosati et al., 2018b). Specifically, buds that form on new mixed 1-year-old shoots (both vegetative and reproductive) can shift between reproductive and vegetative cycle. Therefore, high crop load can delay the elongation of mixed shoots (Proietti and Tombesi, 1996; Dag et al., 2010; Fernández et al., 2015), reducing the presence of new potential reproductive buds for the following year. The influence of crop load on vegetative growth per cultivar and type of shoots was reported on **Table 4**, confirming what has been observed in previous studies. This pattern is evident across all cultivars; Lateral Proleptic shoots of ‘Urano’ cultivar showed the negative correlation (-0.52), highlighting a significant trade-off between vegetative growth and crop load. Similarly, ‘Arbequina’ with Adventitious shoots (-0.42), ‘Coratina’ with sylleptic shoots (-0.35), and ‘Frantoio’ with sylleptic shoots (-0.31) follow the same trend, but with weaker correlations. The other combinations (cultivar x type of shoot) are not reported because they had a correlation less than -0.30. Whitin the same shoot type, shoot length variations due to fruiting need to be considered. The production of shorter shoots can be considered a response to high fruit loads, driven by competition between vegetative and reproductive growth for resources (Rosati et al., 2018b). Additionally, shorter shoots can export carbon sooner, contributing to an earlier carbon redistribution within the plant (Hansen, 1977; Lakso, 1984; Sansavini and Corelli-Grappadelli, 1992; Lauri and Kelner, 2001). GDD showed no correlation with the shoot elongation (L), in line with the results of Ben Sadok et al., 2015 where no effect between the interaction of genotype – environment (GxE) on architectural features was found. Conversely, other studies remark on the influence of temperature and water stress on shoot growth dynamic (Benlloch-González et al., 2024: Zucchini et al., 2023; Siakou et al., 2021) However, the effects of warmer climates on the vegetative phenology of olive trees have received less attention compared to their reproductive development, with even fewer studies examining the influence of different cultivars (Maldera et al., 2024b; Benlloch-González et al., 2024).

**Table 4:** Pearson’s correlation between crop load and shoot elongation (L) per cultivar and type of shoots.

Cultivar	Shoot type	Pearson’s correlation
Arbequina	Adventitious	-0.42
Coratina	Sylleptic	-0.35
Frantoio	Sylleptic	-0.31
Urano	Lateral Proleptic	-0.52

## 4 Conclusions

A logistic regression was applied to modelling the shoot growth dynamics of four self-rooted olive cultivar. Single-phase logistic growth model represented the shoot elongation of olive cultivar. However, ‘Urano’ and ‘Coratina’ cultivar a second growth flush was observed, particularly for lateral proleptic and adventitious shoots. The likelihood of a second vegetative flush may be associated with genetics and physiological traits, highlighting the importance of agricultural management practices (e.g. canopy management, irrigation). Additionally, we observed that GDD had no correlation with shoots elongation. Conversely, crop load showed a negative influence on adventitious shoots of ‘Arbequina’, sylleptic shoots of ‘Coratina’ and ‘Frantoio’ and lateral proleptic shoots of ‘Urano’. Furthermore, ‘Arbequina’ and ‘Urano’ showed the most pronounced *trade-off*, indicating a reduction of shoot length when olive yield was higher. This suggested a different source-sink relationships, probably due to their intrinsic genetic traits as well as, the competition for assimilates between reproductive and vegetative. To our knowledge, this is the first study on the growth dynamics of different olive shoot types for different olive cultivar with distinct levels of vigor, within a SHD orchard. These findings are crucial for understanding cultivar-specific shoot elongation dynamics, supporting the optimization of agricultural practices for a sustainable cultivar-specific management. Further studies need to be defined to study vegetative growth dynamics, including other factors (e.g. Leaf Area Index, trunk diameter, branches length, water stress indices), to improve the olive-growing practices.

## Data Availability

The raw data supporting the conclusions of this article will be made available by the authors, without undue reservation.

## Supplementary material

Supplementary Figure 1 Box plots of residuals at seven time points.

Supplementary Figure 2 Q-Q plot of residuals.

Supplementary Figure 3 Logistic model fit with observed shoot length data.
