# Supplementary figures and images for: Logistic function explains the growth dynamics of different types of shoots of four olive cultivars grown in a super high-density orchard
Source: Front Plant Sci. 2025 Jun 19;16:1542816. doi: 10.3389/fpls.2025.1542816 (PMC12222076; doi:10.3389/fpls.2025.1542816)

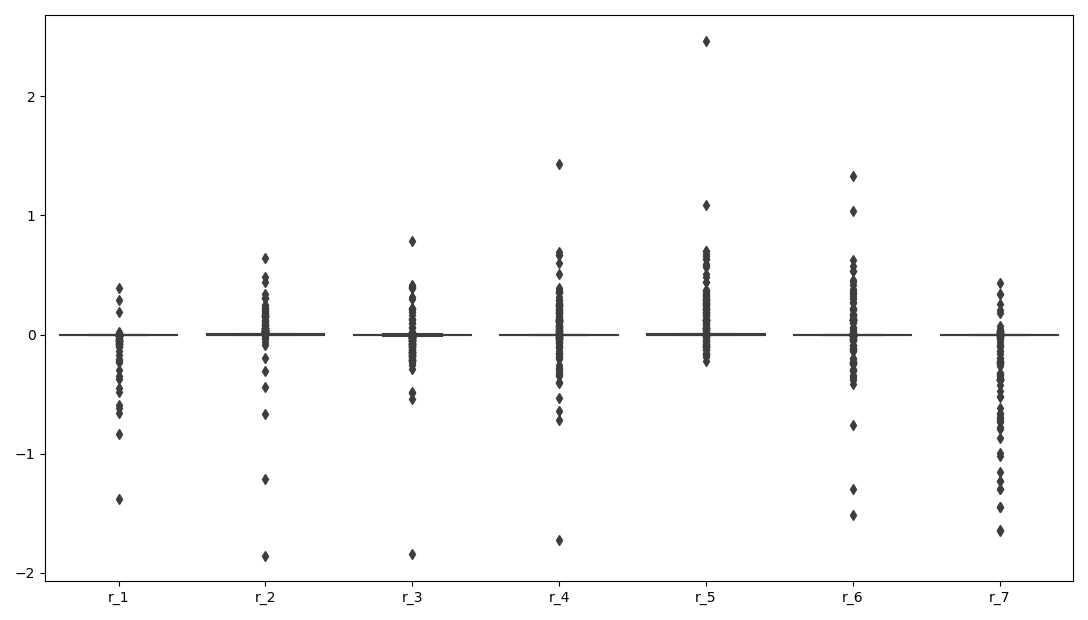

Supplement: Supplementary Figure 1 — Box plots of residuals at seven time points. [file Image1.png]

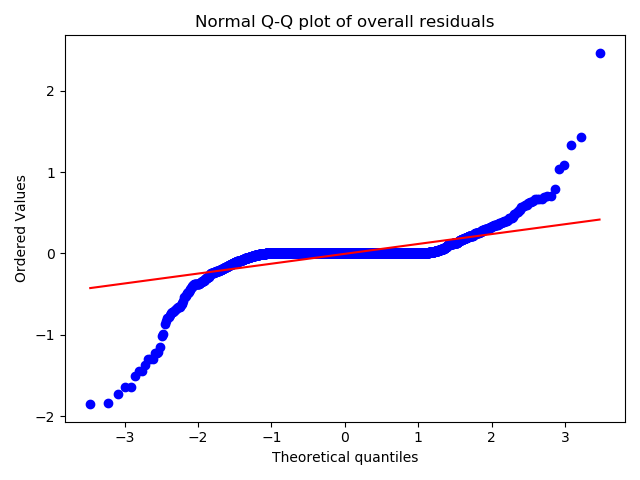

Supplement: Supplementary Figure 2 — Q-Q plot of residuals. [file Image2.png]

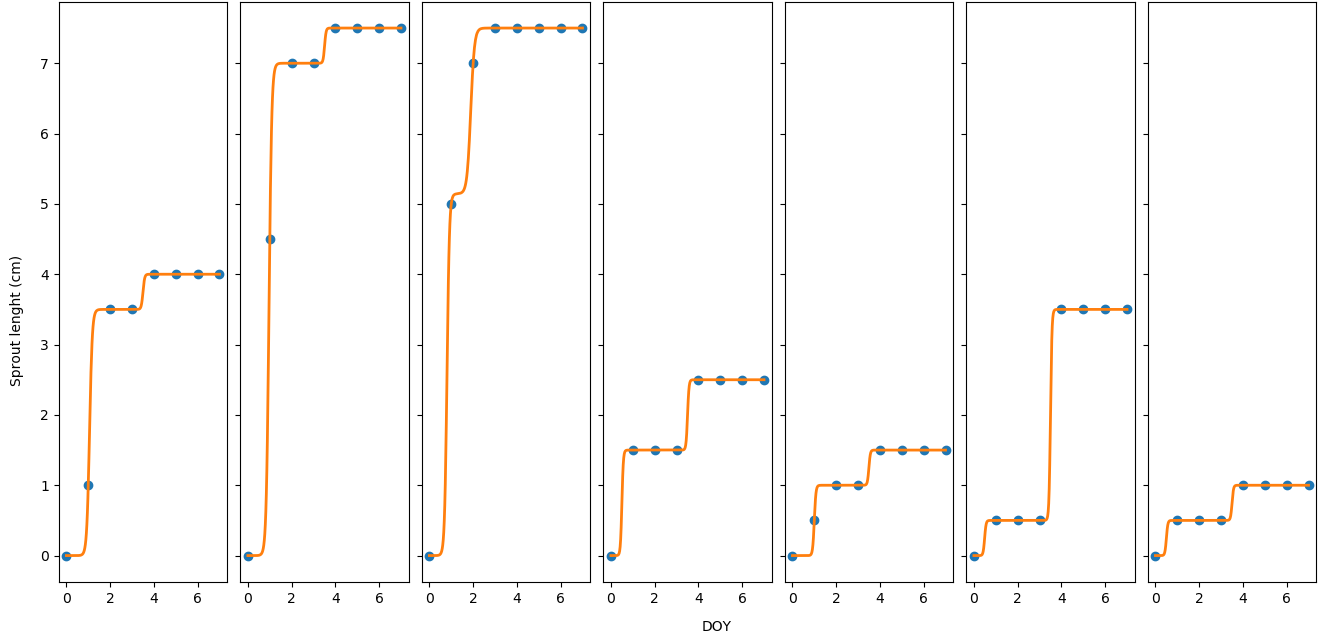

Supplement: Supplementary Figure 3 — Logistic model fit with observed shoot length data. [file Image3.png]
